# Rule-Based Cohort Definitions for Acute Respiratory Failure: Electronic Phenotyping Algorithm

**DOI:** 10.2196/18402

**Published:** 2020-04-15

**Authors:** Patrick Essay, Jarrod Mosier, Vignesh Subbian

**Affiliations:** 1 College of Engineering The University of Arizona Tucson, AZ United States; 2 College of Medicine The University of Arizona Tucson, AZ United States

**Keywords:** computable phenotype, electronic health record, intensive care units, critical care informatics, telemedicine, respiratory

## Abstract

**Background:**

Acute respiratory failure is generally treated with invasive mechanical ventilation or noninvasive respiratory support strategies. The efficacies of the various strategies are not fully understood. There is a need for accurate therapy-based phenotyping for secondary analyses of electronic health record data to answer research questions regarding respiratory management and outcomes with each strategy.

**Objective:**

The objective of this study was to address knowledge gaps related to ventilation therapy strategies across diverse patient populations by developing an algorithm for accurate identification of patients with acute respiratory failure. To accomplish this objective, our goal was to develop rule-based computable phenotypes for patients with acute respiratory failure using remotely monitored intensive care unit (tele-ICU) data. This approach permits analyses by ventilation strategy across broad patient populations of interest with the ability to sub-phenotype as research questions require.

**Methods:**

Tele-ICU data from ≥200 hospitals were used to create a rule-based algorithm for phenotyping patients with acute respiratory failure, defined as an adult patient requiring invasive mechanical ventilation or a noninvasive strategy. The dataset spans a wide range of hospitals and ICU types across all US regions. Structured clinical data, including ventilation therapy start and stop times, medication records, and nurse and respiratory therapy charts, were used to define clinical phenotypes. All adult patients of any diagnoses with record of ventilation therapy were included. Patients were categorized by ventilation type, and analysis of event sequences using record timestamps defined each phenotype. Manual validation was performed on 5% of patients in each phenotype.

**Results:**

We developed 7 phenotypes: (0) invasive mechanical ventilation, (1) noninvasive positive-pressure ventilation, (2) high-flow nasal insufflation, (3) noninvasive positive-pressure ventilation subsequently requiring intubation, (4) high-flow nasal insufflation subsequently requiring intubation, (5) invasive mechanical ventilation with extubation to noninvasive positive-pressure ventilation, and (6) invasive mechanical ventilation with extubation to high-flow nasal insufflation. A total of 27,734 patients met our phenotype criteria and were categorized into these ventilation subgroups. Manual validation of a random selection of 5% of records from each phenotype resulted in a total accuracy of 88% and a precision and recall of 0.8789 and 0.8785, respectively, across all phenotypes. Individual phenotype validation showed that the algorithm categorizes patients particularly well but has challenges with patients that require ≥2 management strategies.

**Conclusions:**

Our proposed computable phenotyping algorithm for patients with acute respiratory failure effectively identifies patients for therapy-focused research regardless of admission diagnosis or comorbidities and allows for management strategy comparisons across populations of interest.

## Introduction

### Overview

Acute respiratory failure occurs in patients that cannot maintain adequate blood oxygen levels (hemoglobin saturation and partial pressure of arterial oxygen), cannot normalize blood pH, or cannot sufficiently compensate for systemic metabolic acidosis. Patients can develop respiratory failure from a multitude of causes, including neurologic injury, toxidromes, musculoskeletal abnormalities, cardiac or pulmonary abnormalities, and sepsis. Conceptually, the treatment of acute respiratory failure involves invasive ventilation or noninvasive ventilation (NIV) strategies. There are multiple modalities for these therapies, and the selection of an intervention depends on the pathophysiologic processes and severity of the disease [1-3]. While noninvasive strategies have been studied among specific patient populations, the various therapies themselves have not been extensively investigated across diverse critical care populations, and there are conflicting data on the efficacy of these strategies [4,5]. Furthermore, given informatics challenges related to electronic health record (EHR) phenotyping such as data completeness, complexity, bias, and accuracy [6], there is a need to clearly define patient cohorts to investigate invasive ventilation and NIV strategies using retrospective EHR data.

The objective of this work was to develop a rule-based computable phenotyping algorithm by ventilation therapy for patients with acute respiratory failure. This allows for characterization and extraction of critically ill patients based on treatment modality beyond the traditional binary classification of ventilation therapy (ie, intubated [invasive] or not intubated [noninvasive]) as well as large-scale application of a rule-based phenotype to a wide range of hospital sizes and types across the United States.

### Background

Clinical management of acute respiratory failure depends on the underlying pathophysiology, but generally can be considered as low-flow oxygen therapy (<15 L/min of oxygen through a nasal cannula, ventimask, or nonrebreathing mask), a NIV strategy that includes high-flow nasal insufflation (15-70 L/min of heated and humidified gas with a titratable fraction of inspired oxygen via a high-flow nasal cannula system) or noninvasive positive-pressure ventilation (via a face mask and ventilator), or invasive mechanical ventilation (via an endotracheal tube [ETT] and ventilator).

While there are multiple NIV modalities [7], we refer to noninvasive positive-pressure ventilation (NIPPV) and high-flow nasal insufflation (HFNI) as two primary NIV strategies. Conventional low-flow oxygen therapy uses traditional oxygen delivery sources to provide supplemental oxygen with flow rates up to 15 L/min. On the other hand, both NIPPV and HFNI are designed to provide either pressure-based or flow-based ventilatory support with titratable respiratory gasses and are therefore considered strategies for noninvasively treating patients with acute respiratory failure [8].

### Significance

NIV strategies are now widely used in an effort to avoid the untoward effects of invasive mechanical ventilation via endotracheal intubation [9,10]. Failure of noninvasive therapy resulting in intubation, however, puts patients at greater risk than those that were intubated without attempting NIV [11-13]. These risks suggest a need for large-scale studies to better understand the use of NIV strategies across specific diagnoses and amongst all patients with de novo acute respiratory failure as well as to identify factors associated with increased risk of NIV failure and opportunities to improve patient outcomes when using these therapies [14,15].

A clinical phenotype, generally defined as a set of observable characteristics representing the current and potentially changing state of a patient [16], is typically developed using diagnosis or other disease-related characteristics. Analysis of ventilation strategies as they pertain to patients broadly, however, is limited. As a result, NIV strategies and subsequent failure that lead to endotracheal intubation are not fully understood across various intensive care unit (ICU) patient populations. Our goal in this study was to address these knowledge gaps by developing a computable rule-based algorithm to identify phenotypes in critically ill patients with acute respiratory failure using retrospective, remotely monitored clinical data.

## Methods

### Data Source

Data were extracted from the eICU Collaborative Research Database. The eICU database is a publicly available critical care telemedicine database containing structured EHR data from ≥200 hospitals throughout the United States from 2014 and 2015 [17]. It includes a wide range of data from basic patient demographics to treatment records, medications, vital signs, and nursing and respiratory therapy notes, all in a structured format. Hospitals contributing to the dataset are from both academic and nonacademic settings and vary in size from 10 beds to 500 beds and by type (eg, medical surgical ICU, cardiothoracic ICU). Data contributions from each hospital depend on site-specific policies, procedures, and interfaces with the remote ICU, or tele-ICU.

### Inclusion and Exclusion Criteria

Inclusion criteria for this study were all adult (≥18 years old) ICU patients with any admission diagnosis or comorbidities with record of invasive ventilation or NIV strategy. All included records required associated time stamps in order to determine ventilation success or failure. Patients were excluded if they were treated using conventional low-flow oxygen or were readmitted to the ICU. Readmissions were excluded to allow for equal comparison of patient outcomes across phenotypes. All inclusion and exclusion criteria were validated by domain experts in respiratory management and critical care medicine.

We developed the phenotypes using a combination of rules and characteristics previously identified by domain experts [18,19] and by first categorizing patients by ventilatory support strategy. For patients where more than one therapy was used, we used time stamps to determine the order in which patients were treated. Our approach consisted of 4 main steps followed by descriptive statistical analysis: (1) systematic exploration of all available structured data and identifying all terms (standardized and nonstandardized) related to mechanical ventilation; (2) identification of patients treated with invasive (intubation) or noninvasive (NIPPV or HFNI) strategies by extracting ventilation-related treatment, medication, and nursing records; (3) treatment record sequencing based on ventilation type as well as start and stop time comparisons to determine which patients failed respiratory therapy; and (4) development of the rule-based phenotyping algorithm in a decision tree format.

### Exploration of Available Data

All available structured data were systematically explored for record types that might indicate ventilation strategy. Of particular interest were nursing charts, respiratory therapy charts, treatment records, infusion drugs and medications, and data pertaining to intraprofessional communication and care planning (eg, variables related to provider type and specialty as well as airway and ventilation status).

Distributions of key terms related to mechanical ventilation were calculated by number of records per term. For example, the term “Intubated/oral ETT” occurred in 59,566 records, while “Intubated/nasal ETT” occurred in 335 records. It is important to note that, in our dataset, these terms are structured data selections and not free-text inputs. Therefore, we were able to search for partial words and phrases (eg, “intub”), which returned all records containing the partial term. Selection of key terms was performed for both invasive and noninvasive ventilatory support. All terms were reviewed by both informatics and clinical experts.

### Identification of Ventilation Therapies

In addition to terms identified in the exploration step, medications related to pre-intubation, intra-intubation, and post-intubation care (eg, rapid sequence intubation medications, neuromuscular blocking agents, and continuous sedative agents) were used to verify invasive mechanical ventilation. Patients in both invasive ventilation and NIV groups were then filtered by the number of repeated records (ie, a patient must have >1 record of each ventilation type to be included in that group). Repeated records and validation across multiple record types were required to minimize the impact of spurious records indicating the wrong type of ventilation in a sequence and misclassifying a patient into another cohort.

### Record Sequencing and Timestamp Validation

Unique patient identifiers were used to identify patients classified in both invasive ventilation and NIV groups. Record timestamps were then used to verify treatment paths of those patients with multiple records of both invasive and NIV. Treatment records were grouped by patient identifier and sorted by record type and timestamp. The difference between invasive and noninvasive timestamps was used to indicate the respiratory therapy sequence for each patient. If NIPPV or HFNI was performed prior to invasive mechanical ventilation, patients were categorized as NIV failure. If NIPPV or HFNI was performed after invasive mechanical ventilation, patients were categorized as having been extubated to NIV.

The timestamps in our dataset are recorded as the number of minutes from ICU admission and may be positive or negative values. For example, an NIPPV timestamp of –90 minutes and an invasive timestamp of 30 minutes indicate that the patient was treated with NIPPV for 90 minutes prior to ICU admission and was intubated 30 minutes after ICU admission resulting in an “NIPPV failure” categorization.

To identify HFNI patients, we used the same approach as for NIPPV with an additional requirement that patients must have record of both noninvasive mechanical ventilation and HFNI. Patients were excluded if there was record of HFNI without record of NIV due to the hierarchical nature of treatment records in the dataset. Failure of HFNI was determined according to the timing sequence relative to invasive ventilation just as with the NIPPV patients. This resulted in 3 HFNI-related groups: HFNI failure patients requiring subsequent intubation, patients treated solely with HFNI with no other form of ventilatory support, and patients extubated to HFNI. Similar to how intubation-related medications were used to validate invasive ventilation patients, structured data from nurse charts were used to validate NIV strategies.

### Defining Phenotypes

All of the described constraints were compiled to create the phenotyping algorithm. The algorithm was constructed sequentially in an easily interpreted decision tree format. Table 1 defines each phenotype and lists the relevant data elements used in the algorithm.

**Table 1:** Phenotypes developed, definition, and the electronic health record data elements used to create and validate the phenotypes.

Phenotype	Definition	Data elements^a^
0: Invasive ventilation	Patients treated with invasive mechanical ventilation only	Treatment records, medications
1: NIPPV^b^	Patients treated with noninvasive positive-pressure ventilation only	Treatment records
2: HFNI^c^	Patients treated with high-flow nasal insufflation only	Treatment records, structured nurse notes
3: NIPPV failure	Patients treated with noninvasive positive-pressure ventilation requiring subsequent endotracheal intubation	Treatment records, medications
4: HFNI failure	Patients treated with high-flow nasal insufflation requiring subsequent endotracheal intubation	Treatment records, medications, structured nurse notes
5: Invasive to NIPPV	Patients extubated to noninvasive positive-pressure ventilation	Treatment records, medications
6: Invasive to HFNI	Patients extubated to high-flow nasal insufflation	Treatment records, medications, structured nurse notes

^a^All data elements extracted with timestamps.

^b^NIPPV: noninvasive positive-pressure ventilation.

^c^HFNI: high-flow nasal insufflation.

### Validation

Algorithm performance was quantified by manually validating a randomly selected 5% of patients in each phenotype. Total accuracy was calculated along with multiclass, microprecision, macroprecision, microrecall, macrorecall, and F1 score. We report the weighted average metrics along with precision, recall, and F1 score of each phenotype individually.

## Results

**Figure 1:**
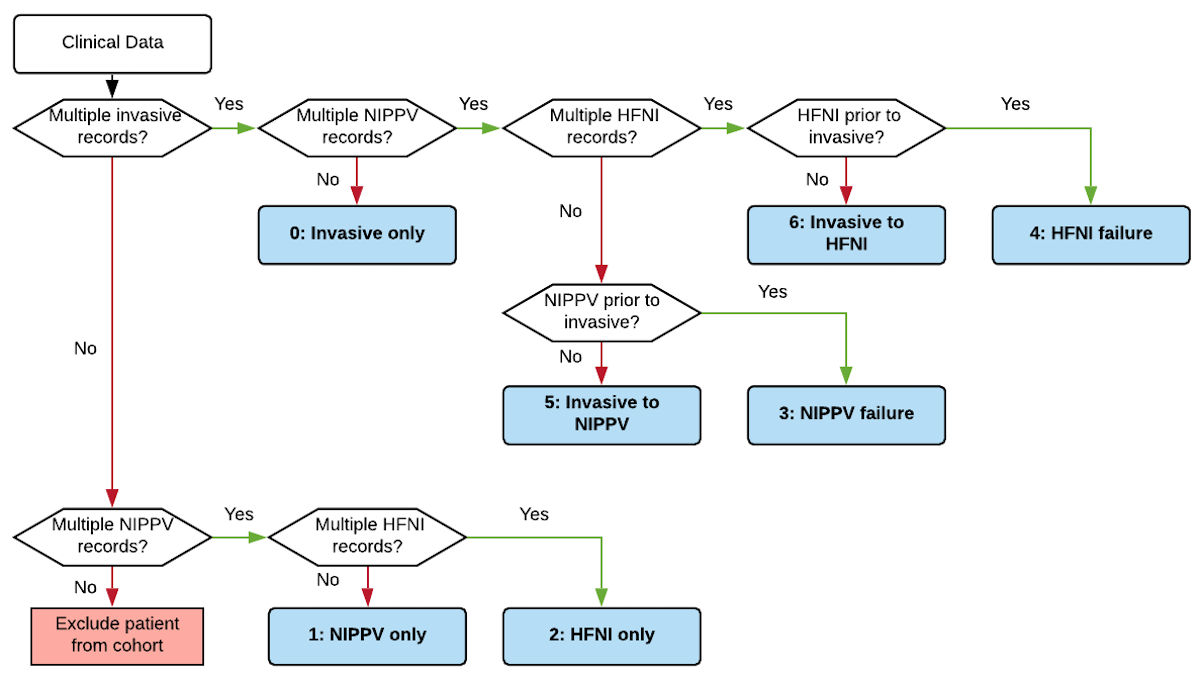
Decision tree model for phenotyping patients with acute respiratory failure. Invasive ventilation patients corroborated by medication records and HFNI corroborated by nurse charts. NIPPV: noninvasive positive-pressure ventilation; HFNI: high-flow nasal insufflation.

Of the 139,367 unique patients in the tele-ICU database, 31,366 were excluded for readmissions. An additional 80,267 patients were excluded for receiving either low-flow oxygen therapy or no ventilatory support. The remaining 27,734 patients were included in the analysis. Using our algorithm (Figure 1), we identified 7 phenotypes based on the ventilation strategy: patients treated utilizing a single strategy (invasive mechanical ventilation, NIPPV, or HFNI), patients that failed NIPPV, patients that failed HFNI, invasive mechanical ventilation patients extubated to NIPPV, and invasive mechanical ventilation patients extubated to HFNI.

### Patient and Data Characteristics

We found that 17,646 of the patients meeting the inclusion criteria were treated initially with invasive mechanical ventilation. Of those, 188 were extubated to HFNI, and 649 were extubated to NIPPV. Patients treated initially with HFNI totaled 1838, of which 636 (34.6%) failed and required invasive mechanical ventilation. Patients treated initially with NIPPV totaled 8250, and 1597 (19.4%) failed, requiring invasive mechanical ventilation. Summary statistics for each ventilation group are shown in Table 2.

**Table 2:** Patient characteristics across phenotypes of invasive and noninvasive mechanical ventilation success and failure.

Patient characteristics		Phenotypes of invasive and noninvasive mechanical ventilation						
		0: Invasive	1: NIPPV^a^	2: HFNI^b^	3: NIPPV failure	4: HFNI failure	5: Invasive to NIPPV	6: Invasive to HFNI
Patients, n (%)		16,809 (60.61)	6653 (23.99)	1202 (4.33)	1597 (5.76)	636 (2.29)	649 (2.34)	188 (0.68)
Age (years), median (IQR^c^)		63.0 (21)	70.0 (20)	72.0 (22)	65.0 (22)	65.0 (23)	66.0 (20)	67.5 (21)
Male gender, n (%)		9895 (58.87)	3336 (50.14)	599 (49.83)	887 (55.50)	347 (54.56)	353 (54.39)	108 (57.45)
**Ethnicity, n (%)**								
	White	13,119 (78.74)	5418 (82.04)	836 (72.07)	1252 (78.69)	430 (68.36)	541 (83.74)	114 (60.96)
	African American	1808 (10.85)	746 (11.30)	104 (8.97)	171 (10.75)	43 (6.84)	42 (6.50)	10 (5.35)
	Hispanic	547 (3.28)	139 (2.10)	135 (11.63)	64 (4.02)	105 (16.69)	25 (3.87)	43 (22.99)
	Asian	199 (1.19)	79 (1.20)	10 (0.86)	16 (1.01)	9 (1.43)	2 (0.31)	2 (1.10)
	Native American	153 (0.92)	28 (0.42)	5 (0.43)	14 (0.88)	2 (0.32)	11 (1.70)	2 (1.10)
	Other/unknown	835 (5.01)	194 (2.94)	70 (6.03)	74 (4.65)	40 (6.36)	25 (3.87)	16 (8.56)
APACHE^d^ score, median (IQR)		69 (41)	57 (29)	56 (28)	75 (38)	72(39)	75 (39)	72 (35.5)
ICU^e^ LoS^f^ (days), median (IQR)		3.23 (4.56)	2.23 (3.07)	2.43 (2.59)	7.48 (9.31)	6.68 (9.10)	5.42 (6.28)	4.93 (5.04)
Hospital mortality, n (%)		3501 (20.83)	1176 (17.68)	123 (10.23)	551 (34.05)	107 (16.82)	135 (20.80)	19 (10.11)

^a^NIPPV: noninvasive positive-pressure ventilation.

^b^HFNI: high-flow nasal insufflation.

^c^IQR: interquartile range.

^d^APACHE: Acute Physiology and Chronic Health Evaluation.

^e^ICU: intensive care unit.

^f^LoS: length of stay.

**Figure 2:**
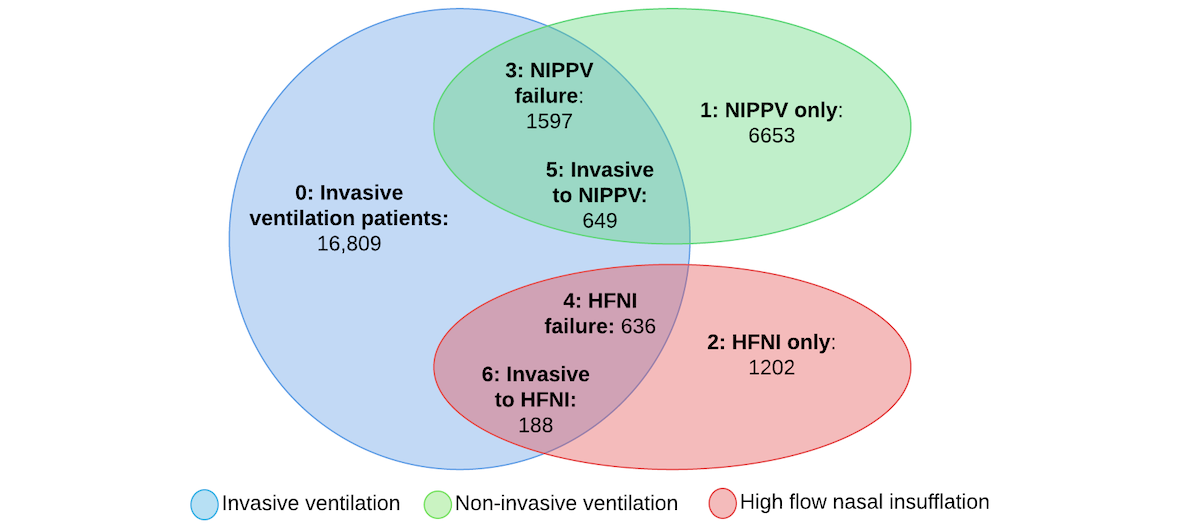
Venn diagram showing the 7 phenotypes based on ventilation therapy. All patient totals are exclusive to each group. Category overlap only indicates patients with multiple record types. For example, 636 patients with HFNI failure are not included in the 1202 patients with HFNI only. NIPPV: noninvasive positive-pressure ventilation; HFNI: high-flow nasal insufflation.

The 7 phenotypes span all ethnicities (although primarily white) and 388 different diagnoses with sepsis, congestive heart failure, and coronary artery bypass grafting among the most common. Figure 2 illustrates the respiratory therapy overlap used to separate the phenotypes based on record sequence, which led to the identification of 2 failure phenotypes (groups 3 and 4) and 2 extubation phenotypes (groups 5 and 6) between invasive ventilation patients with NIPPV and HFNI, respectively.

**Figure 3:**
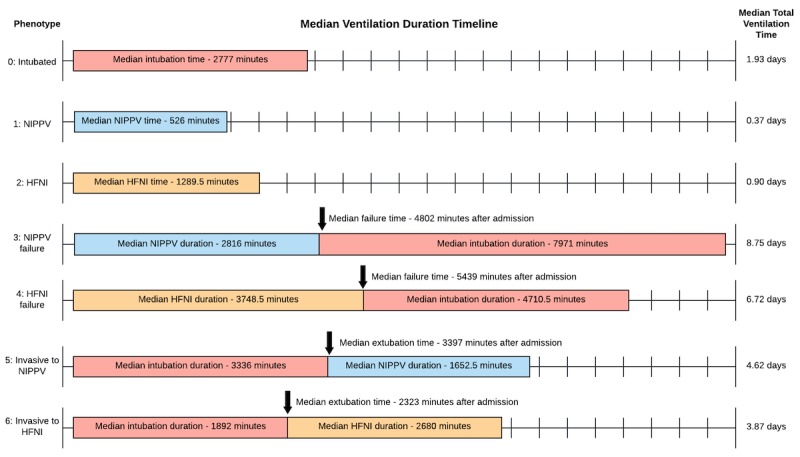
Timeline figure showing median event sequence for patients within each of the 7 phenotypes. Patients that met phenotype criteria but did not have definitive ventilation start and stop times were excluded from the timeline. NIPPV: noninvasive positive-pressure ventilation; HFNI: high-flow nasal insufflation.

The mean ventilation therapy duration for each phenotype is illustrated in Figure 3. Each timeline depicts the ventilation and failure times relative to arbitrary and variable ICU admission and discharge times as event timestamps are labeled as number of minutes from admission. The event sequence remains consistent within each category irrespective of ICU admission time. The failure groups experienced longer total ventilation times than patients treated with one form of ventilation therapy or patients that were extubated to NIPPV or HFNI. Of the 27,734 patients included in our analysis, 7.4% had ventilation start times (intubation or NIV) prior to ICU admission, and 0.81% of NIPPV or HFNI failure times occurred within the first 12 hours (720 minutes) of ICU stay (ie, patients that were brought to the ICU in order to be intubated).

### Validation

Manual validation performed on the randomly selected 5% of records from each phenotype resulted in 1597 patients. The total accuracy across all phenotypes was 88%. The weighted average precision and recall were 0.8789 and 0.8785, respectively, with an F1 score of 0.8599 (Table 3). The NIPPV failure and HFNI failure patients were categorized with accuracies of 73% and 68%, respectively.

The validation process revealed some incorrect classifications between phenotypes. Apparent causes of incorrect classification were: (1) inconsistent definition or use of EHR treatment records; (2) patients with variable, lengthy, and repeated sequences of ventilation records (ie, patient was intubated more than once or attempted NIPPV/HFNI more than once); and (3) erroneous record-keeping typically as a result of continued recording in nursing or respiratory therapy notes of a previous treatment after a patient began an alternative therapy.

**Table 3:** Validation performance metrics for each phenotype.

Phenotype	Precision	Recall	F1 score
0: Invasive	0.9072	0.9365	0.9216
1: NIPPV^a^	0.8617	1.000	0.9257
2: HFNI^b^	0.9846	0.9552	0.9697
3: NIPPV failure	0.7159	0.7326	0.7241
4: HFNI failure	0.4412	0.6818	0.5357
5: Invasive to NIPPV	0.9444	0.6415	0.7640
6: Invasive to HFNI	0.8000	0.0879	0.1584

^a^NIPPV: noninvasive positive-pressure ventilation.

^b^HFNI: high-flow nasal insufflation.

## Discussion

In this study, we effectively used a large, remotely monitored, critical care dataset to define 7 unique therapy-based phenotypes of patients with acute respiratory failure. The phenotyping algorithm is broad enough to potentially be applied to other (bedside or remote) critical care datasets while allowing for therapy-focused research across large and diverse patient populations or mapping to specific disease states, depending on the research question. Developing appropriate phenotypes to analyze respiratory management pathways and clinical outcomes is particularly important for patients that receive more than one strategy, such as NIPPV or HFNI, and then require invasive mechanical ventilation. Failing to identify these phenotypes with granularity can lead to bias in observational studies, where a large proportion of these patients may typically be excluded.

The temporal features used in this study provide increased granularity to expand from 2 (intubated or not intubated) to 7 phenotypes. Multiple record types and repeated measures were used to verify that patients were correctly categorized. Moreover, our iterative algorithm development process that included critical care experts further validates the phenotype results and aligns with lessons learned from previous phenotype validation studies [20,21].

### Standards and Terminology

Our proposed phenotyping algorithm is easily interpreted. Future iterations, however, could be mapped to the Observational Medical Outcome Partnership Common Data Model, allowing for broad use of the phenotype algorithm across different data sources with minimal loss of granularity [22]. Mapping to the Common Data Model could, for example, improve scalability across datasets that may not contain the same terminologies as our dataset with minimal impact to cohort development overall [23,24]. The terminologies, vocabulary, and coding schemas associated with mapping to a standardized data model would then be used in the phenotype algorithm, thus removing potential barriers to widespread application.

Treatment records were the primary identifiers in our algorithm of mechanically ventilated patients, whereas International Classification of Diseases, Ninth Revision and current procedural terminology (CPT) codes could be used for identification of patients or auxiliary verification of correct invasive or noninvasive classification (when codes exist and are present in the EHR). Because there are currently no International Classification of Diseases, Ninth Revision or CPT codes for HFNI, patients must be identified using our phenotype algorithm or a variation thereof.

### Challenges with Noninvasive Ventilation Strategies

It is important to note the hierarchical representation seen in the data regarding NIV. The hierarchy of HFNI as a subcategory of NIV or NIPPV may not be an accurate representation in clinical practice. There is no CPT code for HFNI. Thus, there is no specific guidance relating HFNI to NIPPV in structured data and often no specific order in the EHR, which introduces profound difficulty in identifying and extracting this therapy.

While some clinicians may view HFNI as a lower-level therapy relative to NIPPV (and NIPPV as a lower-level therapy relative to intubation), others may consider HFNI and NIPPV as equal noninvasive strategies. In this phenotyping study, we considered both noninvasive strategies as equivalent alternatives. However, HFNI may be represented differently in other datasets and handled differently among clinicians. Further analysis could determine the proportion of patients treated with both HFNI and NIPPV as a progression in response to improving or worsening patient condition. Therefore, two more theoretical phenotypes exist consisting of patients that fail HFNI and are placed on NIPPV and vice versa. Using our algorithm, however, there were no patients that met those criteria due to the hierarchical structure of treatment records in our dataset.

Free-text record entries were an additional challenge specific to HFNI, namely those in nursing charts. Our dataset primarily consisted of structured data. Nurse chart records that were used for validation of HFNI consisted of sequences of records that ranged from broad to specific that described the record in detail. We filtered nurse charts by oxygen device to find HFNI patients. The next, more specific, entry in the nurse chart record, however, was a free-text entry rather than a predetermined menu selection. Consequently, “high-flow nasal insufflation” had 104 variations, including “HFNC,” “highflow n/c,” “optiflow,” and others, where “NC” generally referred to nasal cannula. This issue was exacerbated with data from ≥200 hospitals; however, the reasons for recording meaningful data are perhaps misunderstood. Individual institutions could benefit from reiterating the importance of consistent recording through policies and standard operating procedures.

Our dataset is inherently limited in that not all hospitals have the same recording interfaces with remote ICU teams [25]. Consequently, patients may be unknowingly misclassified by our phenotyping method. While we account for patients with single erroneous records, data entry mistakes, which was seen to some extent in our validation cohort, would classify patients into incorrect phenotypes. Future iterations of the algorithm should include additional safeguards for correct classification such as inclusion of intubation-related medication timestamps in conjunction with treatment timestamps for further validation. Medications could be separated into pre-intubation, intra-intubation, and post-intubation medications to provide deeper insight into the specific event sequences and used in conjunction with lab and blood gas values. The timestamps associated with these more granular events could improve classification accuracy.

### Clinical Relevance

Our algorithm was developed using a large dataset that included multiple hospitals and thousands of patients. In addition to implications to secondary analyses of EHR data, our algorithm could also serve as a tool for process and quality improvement studies in clinical practice to, for example, analyze and improve resource allocation and workflow in ICUs. However, the work needs validation using other datasets at a health system level (ie, inclusive of patients brought to the ICU to be intubated). The proportion of patients that began NIV prior to ICU admission need further investigation from a clinical viewpoint in order to segregate patients that were transferred to the ICU to be intubated. This would provide greater context to patients who experienced NIV failure, but it was not included in our phenotype algorithm. Rather, the underlying decision making behind intubation could be researched as its own topic using our approach as a tool for cohort development. In addition, patient readmissions to the ICU should be analyzed as a separate cohort, and changes to respiratory management strategy (NIV to invasive and vice versa) upon readmission also need to be investigated using the phenotype algorithm.

It is also interesting to note the disparities in patient characteristics across phenotypes (Table 1), particularly for APACHE severity scores. It is possible that demographics upon admission are influential factors for treatment path decision making. Factors such as age, severity, weight, and comorbidities, for example, may influence clinician decisions as to which patients are good candidates for noninvasive therapies over intubation, although, to our knowledge, defined candidate criteria do not exist widely across institutions.

### Conclusions

Identifying therapy-based computable phenotypes for strategies to treat acute respiratory failure in patients admitted to the ICU is possible using this algorithm, and summary statistics are consistent with previous reports of outcomes in patients that fail noninvasive strategies [26,27]. These phenotypes provide a mechanism for large-scale analyses of factors associated with the risk of failure of NIV strategies — to identify modifiable targets for intervention to reduce those risks. Additionally, we have identified an urgent need for standardized terminologies for noninvasive strategies and record-keeping procedures across institutions.

## Abbreviations

CPT: current procedural terminology.
EHR: electronic health record.
ETT: endotracheal tube.
HFNC: high-flow nasal cannula.
HFNI: high-flow nasal insufflation.
ICD-9: international classification of diseases, version 9.
ICU: intensive care unit.
IQR: interquartile range.
NIV: noninvasive ventilation.
NIPPV: noninvasive positive-pressure ventilation.
